# Chain Conformation and Exciton Delocalization in a Push–Pull Conjugated Polymer

**DOI:** 10.1021/acs.chemmater.3c02665

**Published:** 2023-11-28

**Authors:** Yulong Zheng, Rahul Venkatesh, Connor P. Callaway, Campbell Viersen, Kehinde H. Fagbohungbe, Aaron L. Liu, Chad Risko, Elsa Reichmanis, Carlos Silva-Acuña

**Affiliations:** † School of Chemistry and Biochemistry, Georgia Institute of Technology, 901 Atlantic Drive, Atlanta, Georgia 30332, United States; ‡ School of Chemical and Biomolecular Engineering, Georgia Institute of Technology, 311 Ferst Drive NW, Atlanta, Georgia 30332, United States; ¶ Department of Chemistry and Center for Applied Energy Research, University of Kentucky, Lexington, Kentucky 40506, United States; § Department of Chemical & Biomolecular Engineering, Lehigh University, 124 East Morton Street, Bethlehem, Pennsylvania 18015, United States; ∥ School of Physics, Georgia Institute of Technology, 837 State Street, Atlanta, Georgia 30332, United States; ⊥ School of Materials Science and Engineering, Georgia Institute of Technology, North Avenue, Atlanta, Georgia 30332, United States

## Abstract

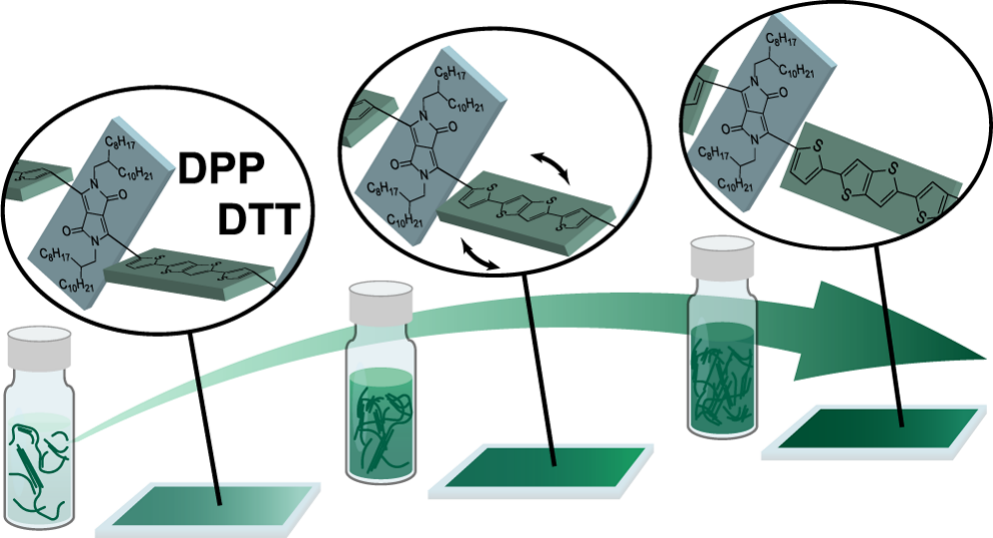

Linear and nonlinear optical line shapes reveal details of excitonic structure in polymer semiconductors. We implement absorption, photoluminescence, and transient absorption spectroscopies in DPP-DTT, an electron push–pull copolymer, to explore the relationship between their spectral line shapes and chain conformation, deduced from resonance Raman spectroscopy and from *ab initio* calculations. The viscosity of precursor polymer solutions before film casting displays a transition that suggests gel formation above a critical concentration. Upon crossing this viscosity deflection concentration, the line shape analysis of the absorption spectra within a photophysical aggregate model reveals a gradual increase in interchain excitonic coupling. We also observe a red-shifted and line-narrowed steady-state photoluminescence spectrum along with increasing resonance Raman intensity in the stretching and torsional modes of the dithienothiophene unit, which suggests a longer exciton coherence length along the polymer-chain backbone. Furthermore, we observe a change of line shape in the photoinduced absorption component of the transient absorption spectrum. The derivative-like line shape may originate from two possibilities: a new excited-state absorption or Stark effect, both of which are consistent with the emergence of a high-energy shoulder as seen in both photoluminescence and absorption spectra. Therefore, we conclude that the exciton is more dispersed along the polymer chain backbone with increasing concentrations, leading to the hypothesis that polymer chain order is enhanced when the push–pull polymers are processed at higher concentrations. Thus, tuning the microscopic chain conformation by concentration would be another factor of interest when considering the polymer assembly pathways for pursuing large-area and high-performance organic optoelectronic devices.

## Introduction

The most fundamental aspect of the materials science of polymer semiconductors concerns the relationship between solid-state microstructure, macromolecular conformation, and the electronic and optical properties of these materials. From a molecular perspective, the optical properties of organic semiconductors are governed by Frenkel excitons,^1^ and this relationship entails the dependence of the exciton optical transition density on the conformation of the polymer backbone, and on the nature of Coulomb coupling between chain segments, both intra- and interchain.^2^ The HJ or photophysical aggregate models, which account for a collective ensemble of interacting transition dipole moments developed by Spano, have been spectacularly successful in quantifying excitonic coupling and the nature of the disordered energy landscape from linear spectral line shapes.^3−10^ These models have been extended to push–pull polymers,^11−13^ in which the role of charge-transfer interactions is evident. In this article, we examine absorption, photoluminescence (PL), and transient absorption optical line shapes in films of poly[2,5-(2-octyldodecyl)-3,6-diketopyrrolopyrrole-*alt*-5,5-(2,5-di(thien-2-yl)thieno-[3,2-*b*]-thiophene)] (DPP-DTT), a push–pull polymer designed for applications in thin-film transistors.^14,15^ The films are cast from solutions of various concentrations; we find that the optical line shapes of the films show a strong dependence on the precursor concentration and correlate the spectral shapes with chain conformation derived from resonance Raman measurements and the corresponding *ab initio* calculations. We conclude that when films are cast from gel-like solutions, excitons are more highly delocalized along the polymer backbone, driven by more planar, less torsionally disordered backbones.

A subtle control of the polymer aggregation could tune the polymer chain conformations, which determine the arrangement of the chromophores. It is crucial to build correlations between the microscopic conformations and photophysical properties. Although previous studies have shown that photophysical and electronic properties of conjugated homopolymers, like poly(3-hexyl)thiophene (P3HT) or polyphenylenevinylenes, are greatly influenced by the long-range order,^7,16−18^ where the exciton dissociation and charge migrations are impacted by the polymeric aggregate fractions, microstructural conformations, defects, etc.,^19−21^ the photophysical aggregates and nonaggregates of short-range order could also play a role in the photogenerated charges.^22,23^ Furthermore, in P3HT-like derivatives, the exciton coherence lengths, which indicate the spatial span of the exciton wave, are varied with different molecular weights.^16^ Specifically, the intrachain exciton extending to more units along the chain backbone in the high molecular-weight P3HTs gives rise to longer PL lifetime, compared to their low molecular-weight counterparts.^17^

In comparison to conjugated homopolymers, the photophysical properties and charge transport behavior might depend on the short-range order more subtly in electron push–pull or donor–acceptor (DA) copolymers.^24−27^ Previous work showed that the electron push–pull nature enhances exciton–exciton annihilation, which gives rise to long-lived bound charge pairs.^28^ Besides, intra- and interchain charge-transfer interactions, representing the wave function overlaps between the electron-sufficient and -deficient moiety along and across the polymer chains, could be subject to the polymer chain backbone orders and π–π stacking, respectively.^12,24,29,30^ Furthermore, the charge-transfer character in the electron push–pull polymers renders a permanent dipole and induces a strong overlap of the electron cloud between push and pull chromophores, which breaks the Kasha approximation of only transition dipole moments interacting.^31^ Therefore, a different energetic landscape from that of homopolymers might be expected in electron push–pull materials.

The impact of inter- and intrachain interactions on the photophysical responses of conjugated polymers has been studied through many ways, such as embedding the diluted semiconductor polymers in inert matrices, or surrounding polymer chains with molecular crowns.^32−37^ In either case, the interchain excitonic interactions are considered to be reduced. However, most studies introduced new components which might lead to ambiguity regarding polymer chain order, microstructures, dielectric environment, and unwanted bath–system interactions. Induced gel formation, on the other hand, only exploits either molecular weight- or concentration-dependence, which are intrinsic properties of polymers in their solution and solid state.^38,39^ Here, we contribute a detailed understanding of the impact of photophysical aggregation on exciton delocalization as well as chain planarization through the gel formation process in push–pull polymers.

## Results

**Figure 1:**
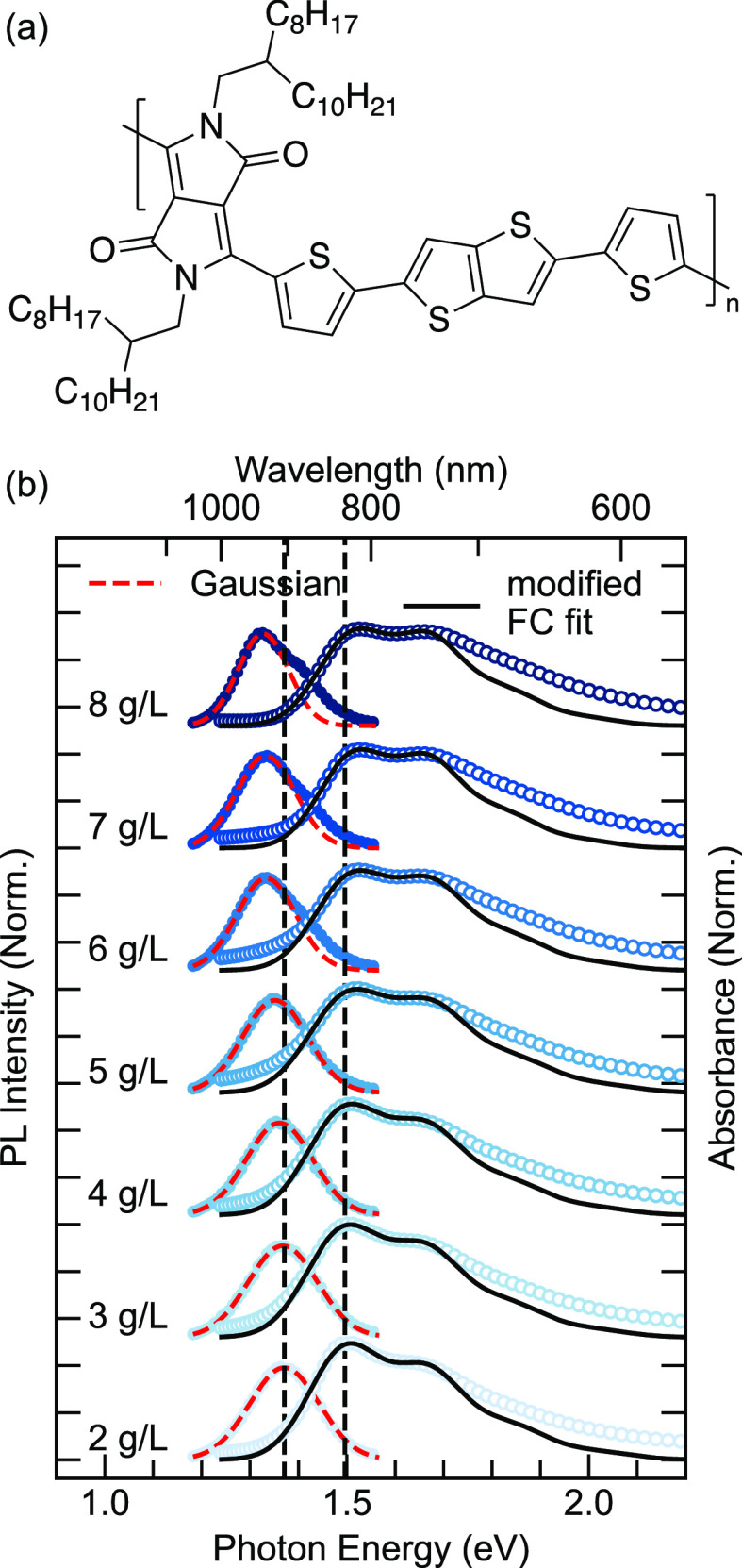
(a) Molecular structure of DPP-DTT. (b) Normalized absorption spectra (open circle) and steady-state PL (solid circle) of thin films deposited from different solution concentrations. We note that the absorption spectra were previously published in ref (40). The PL peaks are fitted with a single Gaussian distribution (red dashed line), while the absorption vibronic replica are simulated with modified Franck–Condon progression (solid black line). The spectra shift for PL and absorption are denoted with dashed black lines with increasing concentrations. A pronounced red shift can be readily observed in the PL spectra; meanwhile, the absorption spectra also display a small blue shift of around 15 meV (see Table S1 in SI for quantitative results).

**Figure 2:**
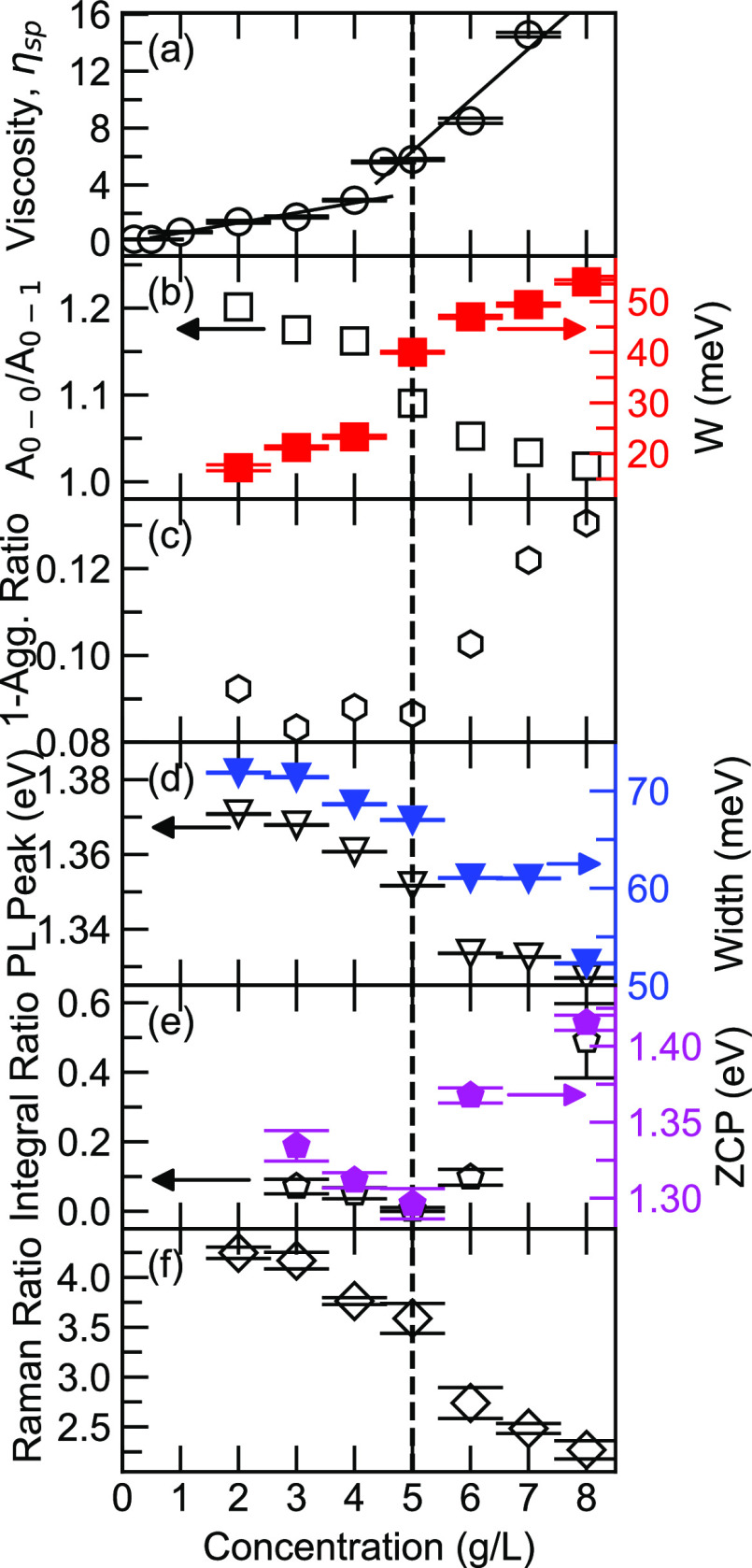
(a) Viscosity measurement on DPP-DTT-chlorobenzene solutions performed at 56 °C, reproduced from ref (40). Coypright 2021 American Chemical Society. The two-regime behavior is visualized by the two linear lines with different slopes. (b) *A*_0–0_/*A*_0–1_ ratio (black open squares) and the effective interchain exciton bandwidth (red solid squares) acquired from FC simulations as a function of concentrations. The error bars associated with exciton bandwidths are also indicated. (c) The ratio of the optical absorption area of the aggregate and nonaggregate spectra as shown in Figure S1 (open hexagon). (d) The peak positions (black open triangles) and widths (blue solid triangles) acquired from Gaussian distributions. (e) The integral ratio (open pentagons) of the shaded area in PIA and GSB, as shown in Figure 4. The zero cross points (ZCPs) are denoted as purple solid pentagons. (f) The ratio of the resonance Raman peak at 1411 and 1366 cm^–1^, with all spectra simultaneously normalized at peak 1525 cm^–1^.The dashed line is the guide for the eye of the critical concentration point.

Using the electron push–pull polymer, as the material of interest displayed in Figure 1a, DPP-DTT thin films were prepared from solutions below and above the viscosity deflecting concentration, c*, as shown in Figure 2a. Below c*, the polymer chains or preassembled small aggregates are more isolated, while they start to pack together with increasing polymer concentration. By leaving the solutions standing still for a few days, the solutions below c* are still fluidic while the ones above c* become immobile as shown in ref (39), Figure S2. The film preparation method was described previously.^40^ To account for the perturbation of the Coulombic interactions within the excitation band, the absorption spectra were fit to a Franck–Condon (FC) progression modified by the contributions of exciton bandwidth, where the effective Huang–Rhys parameter was set to be 0.73 (Figure 1b).^13^ With an increase in concentration, the ratio of the *A*_0–0_ and *A*_0–1_ absorption peaks decreases, which corresponds to an increase in interchain exciton bandwidth from 16 to 54 meV as shown in Figure 2b, accompanied by a small blue shift around 15 meV of the *A*_0–0_ peak (see Table S1 and Figure 1b). The magnitude of the exciton bandwidth falls well under the weakly coupled HJ-aggregate limit; i.e., the estimated excitonic interactions are much smaller than the reorganization energy of the ring stretching mode.^7^ A direct consequence of the larger excitonic interaction in the H-aggregate is an increasing Stokes shift when examining the steady-state photoluminescence (PL) and absorption measurements simultaneously (Figure 1b). Interestingly, in addition to the red shift of the PL peaks, a trend of line width narrowing is also observed as shown in Figure 2d, where the major peak can be simply fitted with a standard Gaussian distribution. Although enhanced interchain excitonic interaction could lead to the red-shifting behavior in the emission of the H-aggregate, it will not result in drastic line narrowing in the PL line shapes. Based on the model developed by Knapp,^41^ Knoester,^42^ and Spano,^6,10^ the line narrowing effects seen in aggregate PL spectra can be explained by the motional narrowing effect, where the distribution of static disorder is averaged out due to the fast-moving excitons. Under Kasha’s approximation, the excited excitons will migrate and are most likely localized at the deepest traps. Therefore, the steady-state PL line shapes would follow the distribution of the deepest traps. Specifically, the distribution of the deepest traps could be impacted by the few following physical factors; the magnitude of static disorder (inhomogeneous broadening line width, σ_*d*_), aggregate size (number of chromophores in each aggregate, *N*), and/or exciton coherence length across chains, in the weakly coupled H-aggregate.^6,10,16^ We interpret these observations as a weakly varying width of the total disorder with processing concentrations. The relevant length scales for the photophysical aggregate are highly microscopic (of the order nearest molecular neighbor), while X-ray crystallography sample length scales are relevant to crystalline order. Nevertheless, we can compare this information with that derived from grazing-incidence wide-angle X-ray scattering (GIWAXS) measurements. Recent measurements on this material^39^ demonstrated invariant *d*-spacing values for π–π stacking of 3.7 Å and lamellar spacing of 19.6 ± 0.2 Å, consistent with other DPP-based copolymers as shown in previous literature.^30,38,43^ Furthermore, consistent full width at half-maximum (fwhm) line widths for each (010) scattering peak are found when comparing all samples, indicating similar paracrystalline static disorder.^44^ To compare the aggregate sizes of the thin films for different concentrations, differential scanning calorimetry (DSC) was performed as shown in Figure S3. A melting temperature of 376 ± 1 °C is found among all samples, and a consistent curve shape for the melting peak is observed. Combining both GIWAXS and DSC measurements, we deduce that the aggregate size and the lattice disorder are probably not the dominant factors for the drastic red shift, as observed in the steady-state PL spectra. Since the intra- and interchain exciton delocalization are countering each other,^16,45^ the motional narrowing effect could be ascribed to the variation of the exciton dispersion due to the change of polymer chain order in the HJ-type photophysical aggregates, as will be demonstrated later on.

Another distinctive feature from the PL spectra is the emerging side peak on the high energy shoulder when the concentration crosses c*. As it is separated from the dominant Gaussian peak by 100 meV, we exclude the possibility of it being a vibronic satellite of the typically dominant 160–180 meV modes. A similar trend is also observed within the absorption spectra (Supplemental Figure S1); the deviation from the aggregate spectra on the high-energy side (from 1.43 to 1.68 eV) is partially ascribed to the absorption of the nonaggregates.^7,43^ Although the ratio of the extinction coefficients between the aggregates and nonaggregates is unknown, we can still deduce the change of the component ratio by assuming the molar extinction coefficient ratio being constant. As there is an increase in the optical absorption from the nonaggregates to the aggregates spectra, the relative amount of nonaggregates is supposed to increase. A constant ratio is observed before c* while the relative contribution from the nonaggregates starts to increase drastically after the critical concentration, as shown in Figure 2c. Therefore, the new PL side peaks can be correspondingly assigned to the emission of such photophysical nonaggregates.

**Figure 3:**
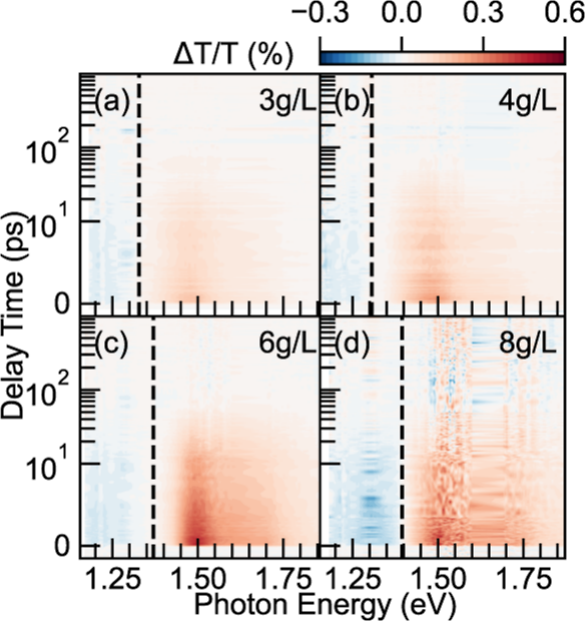
Transient absorption spectral maps showing the differential transmission signals of (a) 3, (b) 4, (c) 6, and (d) 8 g/L samples pumped with a 760 nm pulsed beam under 5 μJ/cm^2^ fluence. The transmission PIA and GSB signals are colored blue and red, respectively. The dashed line lies in the zero-amplitude cross points for each sample, where a clear shift is observed.

**Figure 4:**
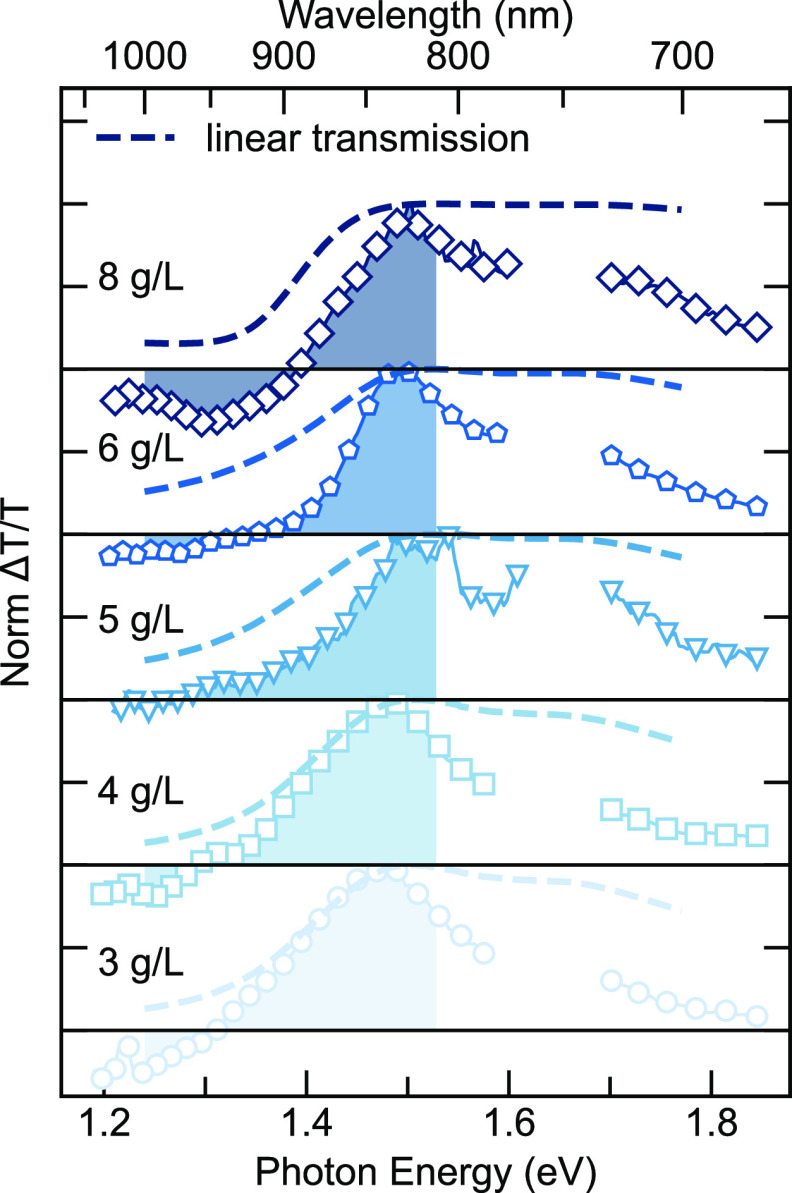
Normalized transient absorption spectra integrated from 0.15 to 1.0 ps measured for samples of 3, 4, 5, 6, and 8 g/L. The cutoff from 1.57 to 1.71 eV is due to the leak of pump beams. The shaded area is integrated to estimate the ratio between GSB and PIA. The differential transmission spectra are overlapped with the linear transmission spectra (dashed line) from Figure 1.

The contributions of the nonaggregate were probed via transient absorption (TA) spectroscopy across the viscosity deflecting concentration. In Figure 3 we display TA spectra for 3, 4, 6 and 8 g/L films, with the pump pulse centered at 760 nm, and with fluence under 5 μJ/cm^2^. (Measurement for films prepared from 5 g/L solutions are presented in Figures S4 and S6 in the Supporting Information.) We note that local heating of the laser beam could lead to a change in the microstructure of the aggregates. Within the fluence range, we observe consistent line shapes as shown Figure S6. However, the exact effects of local heating on the microstructural changes are worth further investigation in this material system. The four TA plots show two domains where the red and blue represent the ground state bleaching (GSB) and photoinduced absorption (PIA), respectively (Figure 4). All subplots show a dominant GSB signal, and the high absorption coefficient is commonly observed due to the long persistence length of the conjugated copolymers.^46^ The lifetimes of GSB are around 18 ps (see SI Figure S7). The PIA signals, on the other hand, show a comparable lifetime to that of the GSB, which suggests that the high-lying excited states relax to the lowest excited vibronic state on a comparable time scale. It is worth mentioning that since we adopted relatively low pump fluences here, the signals become less clean when the time delay is beyond 100 ps, which is specifically emphasized in Figure 3d. However, as shown in Figure S5, the spectral cuts at longer time delays (beyond 100 ps) show noisy features, most likely due to the low fluences we applied. It is unknown at this point if some of the features are real but further investigations on possible thermal photo products will be interesting.

To avoid arbitrary spectral drifting at early times, the spectra were averaged by taking the temporal cuts from 0.15 to 1 ps as displayed in Figure 4. Within the polymer films deposited from higher concentrations, a new excited-state feature emerges in the PIA region. The zero cross points, defined as photon energies where the differential transmission signal is zero, indicate counteracting contributions between the positive signal (GSB) and the negative signal (PIA). The zero cross points (black circles) with respect to the concentration are plotted in Figure 2e. The zero cross points are observed to have a slight decrease up to c*, followed by a drastic increase. The measurement of the 5 g/L film shows a clear small peak around 1.33 eV (932 nm) due to stimulated emission (SE), which also gives rise to the red shift of the zero cross points in addition to GSB. As noted, the zero cross points for samples of 4 and 6 g/L might also have contributions from SE, even though they are much weaker than the 5 g/L sample. As the precursor’s concentration increases, the new growing PIA feature starts to mask the SE signal as they are located at the same wavelength. The overlaps between SE and PIA result in such unusual behavior of the shift of zero cross points. The relative integral ratio of the PIA feature and the GSB of the excitons shows a similar trend by comparing the integrals of the absorption in these two regions (shown as the shaded area in Figure 4). As mentioned above, the turning point is also displayed around the critical concentration, c*, from where there is an increase in the optical absorption of the nonaggregates content. Therefore, a direct absorption from the excited states in the nonaggregates could contribute to this enhanced PIA signature, which is consistent with a growing content of nonaggregates in samples prepared from high concentrations. On the other hand, it is also worth pointing out that the new PIA features resemble the derivative-like Stark effect features,^47,48^ as the differential spectra are perturbed by the induced electric field from the accumulating photogenerated charges at the interfaces of aggregates and nonaggregates.^22^ The degenerate states are lifted by the induced electric field to give such a new feature, especially when comparing the differential line shapes with the linear transmission spectra. Below c*, the GSB transition very much follows the linear transmission, while above c*, the differential spectra have a sharper transition. The two factors mentioned above could both contribute to the new PIA features.

**Figure 5:**
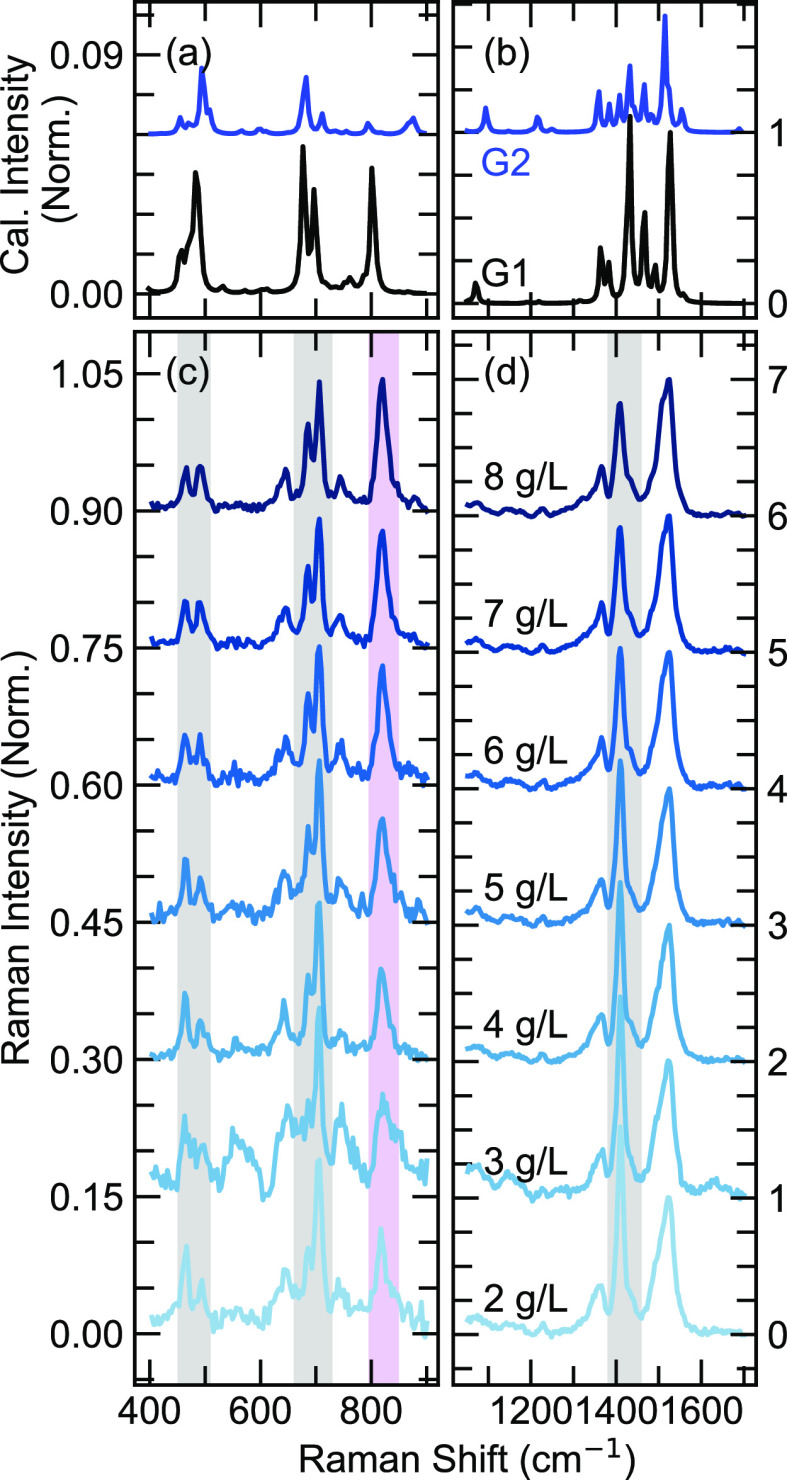
(a, b) Calculated resonance Raman spectra with geometry 1 (black) and geometry 2 (blue), respectively; both are normalized against the geometry 1 peak intensity at 1525 cm^–1^. The spectra are normalized by their relative calculated maximum intensity. (c, d) Experimental resonance Raman spectra displayed within both low and high Raman shift ranges. All experimental spectra are simultaneously normalized at 1525 cm^–1^, which is a C=C stretching mode localized in the DPP unit. The peaks shaded in gray indicate a decrease in Raman intensity, while the peaks shaded in purple indicate increasing Raman intensities with concentrations.

To further understand the polymer chain order and local conformations, we performed resonance Raman spectroscopy to study the on-chain vibrational modes, which are coupled to the electronic transitions. As a more torsionally ordered polymer chain backbone could sustain a longer exciton coherence length,^16^ a more delocalized electron density would weaken the resonance Raman intensity.^17,49^ Here, the high-energy band in DPP-DTT at 488 nm was excited, which contributes to a delocalized π–π* transition as demonstrated by Wood et al.^49^ The Raman spectra are shown in Figure 5c,d. Interestingly, we observed no shifts of the Raman modes, while the intensities of certain modes vary substantially. The most significant change is observed at 1410 cm^–1^ within the high-frequency range from 1200 to 1600 cm^–1^ displayed in Figure 5d, whereas the more substantial variations occur in the low-frequency range as shown in Figure 5c.

The Raman-active modes are assigned based on density functional theory (DFT) calculations. These calculations were performed on a symmetric DPP-DTT trimer truncated with a fourth DPP unit and a terminal thiophene unit, as shown in Figure S8. The additional DPP-T unit is utilized to allow for a symmetric charge distribution. The calculated Raman spectra are displayed in Figure 5a,b. As demonstrated by Chaudhari et al.,^30^ two different geometries might coexist in the ensemble, both contributing to the Raman cross section. In geometry 1 (G1), the oxygen atoms of the DPP unit are oriented close to the sulfur atoms of the neighboring T units, while in geometry 2 (G2), they are instead oriented near the hydrogen atoms of the neighboring T units. The optimized structures of G1 and G2 are shown in Figure S8. Based on DFT calculations, the ground-state energy of G1 is lower than that of G2 by approximately 41.2 kJ mol^–1^. The calculated Raman spectra of both geometries are shown in Figure 5; both spectra are normalized to the peak at 1525 cm^–1^ in G1. In the following discussion, all Raman shifts refer to experimental results, unless specified otherwise. A complete assignment of the Raman modes is presented in Table 1.

**Table 1:** Comparison between the Experimental and the Simulated Resonance Raman Modes^a^

		Norm. intensity		
Exp. Raman shift (cm^–1^)	Simulated Raman shift (cm^–1^)	2 (g/L)	8 (g/L)	Qualitative assignment^d^
1525	1531	1^b^	1^b^	L: 1,4-DPP, L: asym. on 1,3-DTT
	1525			L: 2,3-DPP , L: sym. ring deformation on 2-DTT
1491	1493	0.507 ± 0.014	0.363 ± 0.009	D: gentle DTT and DPP ring deformation, asym. m-Th ring deformation
1410	1431.9, 1432.0^c^	1.507 ± 0.007	0.841 ± 0.023	D: 1,2,3-DTT ring deformation due to
1366	1383	0.355 ± 0.004	0.371 ± 0.009	L: 2,3-DPP
	1381			L: 4-DPP
818	802	0.103 ± 0.011	0.135 ± 0.001	D: sym. on 1,2,3,4-DPP units
706	698	0.190 ± 0.010	0.141 ± 0.004	D: gentle ring breathing of 1,2,3-DTT unit and 2,3-DPP unit
686	678	0.095 ± 0.008	0.096 ± 0.001	
643	—	0.072 ± 0.003	0.038 ± 0.001	—
494	482	0.045 ± 0.010	0.048 ± 0.004	D: torsion of 1,2,3-DTT units and ending m-Th units
	471			
467	455, 456^c^	0.085 ± 0.010	0.044 ± 0.004	D: ring deformation of 1,2,3-DTT units due to the stretching of S atoms

a Simulation results correspond to DPP-DTT geometry 1.

b The whole spectrum is normalized at 1525 cm^–1^.

c Essentially degenerate modes.

d L: local, D: delocalized. 1, 2, 3, and 4 label the order for DPP or DTT unit displayed in Figure 6; *C*_*t*_, *C*_*s*_, *C*_*p*_ are ternary, secondary, and primary carbon atoms, respectively; b-C: bridgehead carbon of polycyclic rings; m-Th: monomeric thiophene ring.

To allow us to compare the change in Raman intensities quantitatively among different samples, a vibrational mode that is not significantly influenced by the torsional order of the polymer backbone (i.e., a local or intraunit vibrational mode) is used as a benchmark. Herein, such a mode is chosen to be the local C=C stretching in the highly rigid DPP unit at 1525 cm^–1^, coupled with a local, asymmetric DTT ring deformation, as shown in the vector diagram in Figure 6a. Such a localized C=C stretching mode on the DPP unit is also observed in a related DPP-based copolymer.^49^ Another localized stretching mode at 1366 cm^–1^ associated with the two bridgehead carbon atoms on the DPP units shows a constant intensity among all samples, supporting our rationalization. In contrast, the greatest changes in intensity are observed at 1416 cm^–1^, where the DTT ring demonstrates strong deformation as shown in Figure 6b. Interestingly, the corresponding simulated Raman peaks are calculated to be doubly degenerate, with one mode being the ring deformation of the second and third DTT units and the other mode being that of the first and fourth DTT unit. Such “globally” delocalized DTT ring deformation directly contributes to a more delocalized exciton wave along the polymer chain backbone, leading to a weaker vibronic coupling strength and decrease of the Raman intensity. A more quantitative demonstration is displayed in Figure 2f, where the intensity ratio of Raman peaks at 1410 and 1366 cm^–1^ is shown to decrease, with clear two-regime behavior.

**Figure 6:**
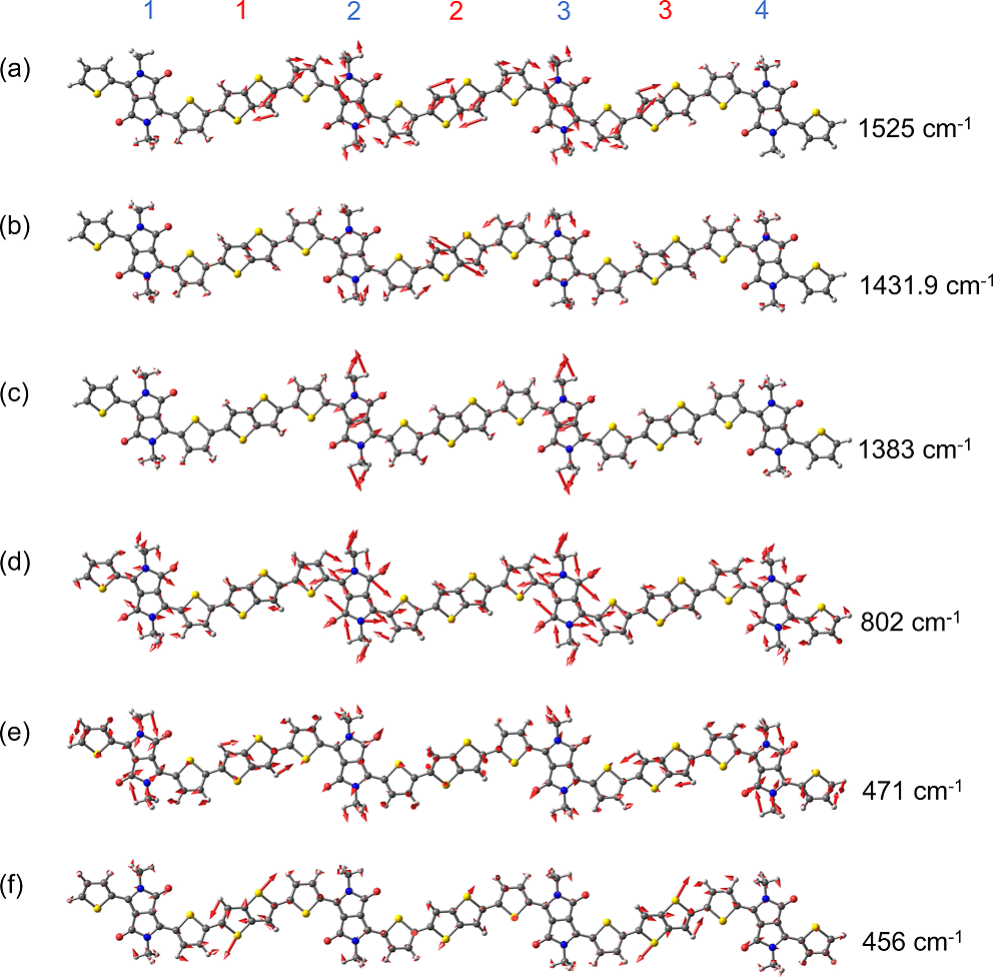
Vector diagrams for the Raman modes of a symmetrically truncated DPP-DTT trimer in geometry 1. (Raman modes for geometry 2 are shown in Figure S9.) Alkyl side chains are substituted with methyl groups for computational efficiency. Atomic color scheme: gray = carbon, white = hydrogen, yellow = sulfur, blue = nitrogen, red = oxygen. The blue and red numbers at the top indicate the indices of the DPP and DTT units, respectively. Atomic displacements are indicated by the arrows.

The other two globally delocalized vibrational modes, at 706 and 467 cm^–1^, show a similar decreasing trend in intensity, where the former mode is a gentle ring breathing motion among all DPP and DTT units and the latter is a degenerate ring deformation mode in all DTT units (Figure 6f). Aside from the stretching modes, the dynamically disordered thienothiophene unit also experiences a strong torsional motion, as observed at 494 cm^–1^ (Figure 6e). Besides the two different trends and their associated vibrational modes, an intriguing increasing trend is observed at 810 cm^–1^, colored purple in Figure 5c. This mode corresponds to the global symmetric C–N stretch in the DPP units (Figure 6d), which localizes the exciton wave. Therefore, when the polymer chain backbone becomes more planarized, which sustains a more delocalized exciton, the vibrational mode at 810 cm^–1^ is not only able to collect the electron density but also redistribute that density in an almost perpendicular direction relative to the polymer chain. The final result is an increase in intensity for higher concentration samples.

Such observation of the Raman intensity variations aligns well with the observation of the PL motional narrowing effect, as mentioned above, indicating improved polymer chain planarization in samples prepared at concentrations higher than the gel formation concentration. Besides, the detailed analysis of the absorption and PL spectra shows the increased exciton bandwidth, indicating stronger interchain interaction. Another surprising finding of higher optical contributions from the nonaggregates with increasing concentrations surfaces when comparing the line shape and oscillator strength from the linear absorption and nonlinear TA measurements. The strong correlations between the viscosity and spectroscopic results can now be well established (Figure 2).

## Discussion

Electron push–pull polymers, unlike conjugated homopolymers, exhibit strong charge-transfer character between the electron sufficient and deficient units within chains. This intrachain interaction plays a dominant role in the optical spectral structure of the material, particularly in enhancing the intrachain (J-like) coupling of chromophores versus interchain (H-like) coupling. The spectral features that arise from J-like coupling, namely the enhancement of the 0–0 spectral peak intensity in the absorption and PL vibronic progression, are attributed to the in-phase electron and hole integrals, and are prevalently shown in other electron push–pull polymers.^46,50−53^ As further demonstrated by Chang et al., the dominant interchain charge transfer interaction in DPP-based systems could lead to abnormal red-shifted H-type aggregate behavior, when the electron and hole transfer integral is out-of-phase, which has been shown experimentally.^13^ While it can be established that the sign of charge-transfer integrals is sensitive to the donor/acceptor stacking arrangement, i.e., π–π stacking geometry and distance,^54^ the investigation of the stacking arrangement on a molecular/atomic level requires rigorous two-dimensional solid-state nuclear magnetic resonance spectroscopic studies, which to date have been limited for push–pull polymers especially in thin films.^30,55−57^ Of particular relevance, Chaudhari et al.^30^ showed that DPP-DTT polymer aggregates adopt a geometry where the donor and acceptor units between chains are alternating stacked when the films are prepared by spin coating. However, they also point out that a segregated donor-on-donor or acceptor-on-acceptor stacking arrangement might have slightly lower energy compared to the slip-on geometry. Such a stacking order could be achieved when certain processing conditions are met. We posit that the fraction of chromophores that adopt such arrangements varies strongly with processing conditions and with a resulting microstructure that is responsible for the trends with concentration reported in Figure 2.

Under the influence of the solution concentration, the resonance Raman spectra displayed the most changes in the low-frequency range, where most of the contributions originate from the stretching and torsional modes of the DTT unit. Such conformational disorder stems from the rotational invariance of the mesogenic groups (i.e., thienothiophene component), and the favorable lowest-energy backbone conformation could be adopted easily.^58^ Indeed, previous DFT calculations performed on DPP-DTT have shown that the different conformations of the DTT unit (e.g., different twisting angles between the thienothiophene and monomeric thiophene rings) have more minor energy difference, compared to that of the DPP unit, implying that the disorder source possibly originates from the former.^30^ In contrast, the DPP units are highly planarized and rigid, as verified by the localized vibrational modes seen in Figure 5d in the high-frequency region. Note that whether the Raman intensities increase, decrease, or remain constant depends on the nature of each vibrational mode; some of the thienothiophene ring deformations along the chain backbone will aid in dispersing the exciton wave, while specific DPP ring deformation modes will lead to exciton wave localization, perpendicular to the polymer chain backbone. This direction dependency reflects the tensor aspects of the Raman polarizability. The large repeat unit will have not only diagonal elements but also off-diagonal elements in the polarizability expression.

Current studies do not allow us to quantitatively determine the spatial correlation of site energies, which can be quantified by the *I*_0–0_/*I*_0–1_ PL ratio,^16^ specifically, due to limitations in the PL detection range. However, as mentioned earlier, the 0–0 peaks become more red-shifted and narrower with increasing concentration. Furthermore, from the resonance Raman spectra, we can conclude that a more dispersed exciton wave is achieved along the polymer chain backbone with increasing concentration. Therefore, it is reasonable to deduce that the polymer chain backbone is more planarized when the processing concentrations increase. Combining both observations, we formulate the hypothesis that the more dispersive exciton wave along the polymer chain leads to a greater extent of spatial correlations of energies, which was described in the J-aggregates as shown by Knapp^41^ and Knoester.^42^ It is worth noting that the spatial correlation function of site energies should be two-dimensional, both along the polymer chain backbone and across the chains in the π-stack, which was demonstrated in P3HT by Spano et al.^10,16,59^ Specifically, in the high *M*_*w*_ P3HT, the extent of exciton coherences along (across) the chains is higher (lower) than that of low *M*_*w*_ P3HTs.^16^ To accurately account for the exciton coherences of DPP-DTT, rigorous calculations of the two-dimensional correlation functions and a larger range of PL measurements are needed.^16^

## Conclusions

We demonstrate that the exciton in push–pull polymers is more delocalized in films cast from solutions in which the concentration surpasses the viscosity threshold for gelation. We hypothesize that this phenomenon is attributed to enhanced chain backbone planarization, as indicated by resonance Raman spectroscopy and DFT calculations. Analysis of the absorption and steady-state PL spectra is consistent with a more highly delocalized exciton along the chain backbone, and more chromophores uncoupled from photophysical aggregates are also formed above the gel formation concentration. The contributions to the transient absorption spectra at the time scale of a few picoseconds are likely 2-fold: derivative-like spectral line shapes due to accumulating photogenerated charges at the aggregate/nonaggregate interfaces^17^ and direct excited-state absorption from the nonaggregate chromophores. We demonstrate the importance of understanding the short-range polymer chain order and excitonic interactions. Manipulating such short-range length scales could further our understanding of preaggregate assembly and favorably accelerate the development of next-generation organic optoelectronics.

## Experimental Methods

### Sample Preparation

DPP-DTT (*M*_w_ = 290,000 g mol^–1^, dispersity = 2) was purchased from Ossila Limited. For sample preparation, a stock solution of 10 g/L DPP-DTT in chlorobenzene (anhydrous, Sigma-Aldrich) was prepared by heating at 100 °C for around 4 h, followed by heating at 60 °C overnight. Then solutions with lower concentrations are diluted from the stock solutions. The DPP-DTT thin films are prepared by wire-bar coating the solutions of different concentrations on fused-silica substrates at 56 °C, followed by annealing for 10 min.

### Vis–NIR Absorption Spectroscopy

The vis–NIR absorption measurements were performed using Cary 5000 UV–vis–NIR spectroscopy.

### Photoluminescence Spectroscopy

The steady-state photoluminescence spectroscopy is performed using the inVia Renishaw Spectrometer in the backscattering configuration. The samples are illuminated by a 785 nm red laser.

### Ultrafast Transient Absorption Spectroscopy

The transient absorption measurements are performed using an ultrafast laser system (Pharos Model PH1-20-02-10, Light Conversion). Tunable wavelengths are generated using a laser fundamental of 1030 nm at a 100 kHz repetition rate. The integrated transient absorption was measured in a commercial setup (Light Conversion Hera). The wavelengths of the pump can be tuned from 360 to 2600 nm by feeding 10 W laser output to a commercial optical parametric amplifier (Orpheus, Light Conversion, Lithuania) while the probe beam is generated by sending 2 W to a sapphire crystal to obtain a single-filament white-light continuum in the spectral range of 490–1060 nm. The probe beam was collected by an imaging spectrograph (Shamrock 193i, Andor Technology Ltd., U.K.) coupled with a multichannel detector (256 pixels, 200–1100 nm wavelength range) after being transmitted through the sample. All of the samples were measured in a homemade vacuum chamber.

### Resonance Raman Spectroscopy

The resonance Raman spectra are measured using the inVia Renishaw Raman spectrometer, where the samples are excited with a 488 nm laser with a backscattering configuration.

### Quantum Chemistry Calculations

Density functional theory (DFT) and time-dependent DFT (TDDFT) calculations were performed at the LC-ωHPBE/6-311G(d) level of theory using the Gaussian 16 Rev. A.03 software suite.^60^ Empirical gap tuning was performed for the two oligomer geometries following the method of Sun et al.,^61−65^ obtaining converged range-separation parameters of ω_1_ = 0.1295 (for G1) and ω_2_ = 0.1216 (G2). Following optimization, vibrational frequency analysis was performed on the oligomer with vibrational scaling factors of 0.995 (G1) and 0.968 (G2) applied to the calculated Raman frequencies. The Raman activities were converted to intensities consistent with prior literature;^66,67^ further details are available in the Supporting Information. A TDDFT calculation was performed to obtain the excitation wavelength, 439 nm (22780 cm^–1^), corresponding to the wavelength used in the associated experimental spectroscopy.

## Supporting Information Available

Complete modified FC analysis of the absorption spectra, Gaussian fit of the steady-state PL spectra, DSC thermograms, TA maps measured under the lowest and highest fluences, GSB and ESA signal magnitude dependence upon, and double-exponential-decay fits for the kinetic traces (PDF)
