# Supplementary material for: Chain Conformation and Exciton Delocalization in a Push–Pull Conjugated Polymer
Source: Chem Mater. 2023 Nov 28;35(23):10258–67. doi: 10.1021/acs.chemmater.3c02665 (PMC10720347; doi:10.1021/acs.chemmater.3c02665)
Supplement: Supplementary file 1 — cm3c02665_si_001.pdf [file cm3c02665_si_001.pdf]

Supporting Information: Chain Conformation and Exciton Delocalization in a Push-Pull Conjugated Polymer

Yulong Zheng,[†] Rahul Venkatesh,[‡] Connor P. Callaway,[¶] Campbell Viersen,[†] Kehinde H. Fagbohungbe,[¶] Aaron L. Liu,[‡] Chad Risko,[¶] Elsa Reichmanis,[§] and Carlos Silva-Acuña[*,†,‖,⊥]

†*School of Chemistry and Biochemistry, Georgia Institute of Technology, 901 Atlantic Drive, Atlanta GA 30332, United States*

‡*School of Chemical and Biomolecular Engineering, Georgia Institute of Technology, 311 Ferst Drive NW, Atlanta GA 30332, United States*

¶*Department of Chemistry and Center for Applied Energy Research, University of Kentucky, Lexington, Kentucky 40506, United-States*

§*Department of Chemical & Biomolecular Engineering, Lehigh University, 111 Research Drive, Bethlehem PA 18015, United States*

‖*School of Physics, Georgia Institute of Technology, 837 State Street, Atlanta GA 30332, United States*

⊥*School of Materials Science and Engineering, Georgia Institute of Technology, 771 Ferst Drive NW, Atlanta GA 30332, United States*

E-mail: carlos.silva@gatech.edu

Absorption and Steady-state PL

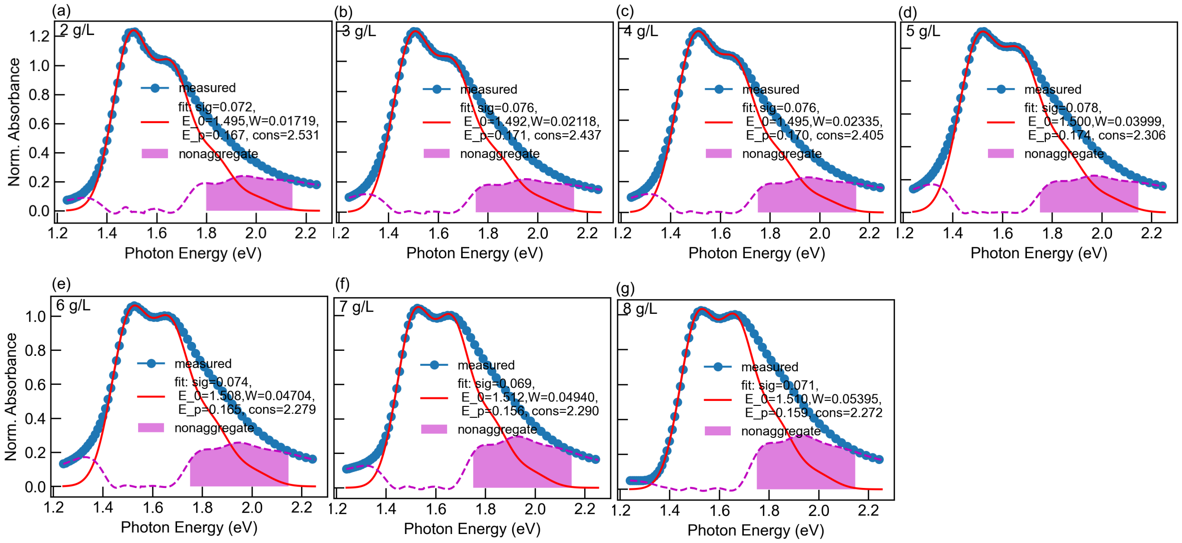

Figure S1: (a)-(g) Modified FC fit for all absorption spectra of the thin films prepared from different concentrations. The parameters are shown in the main article Table 1. Except for the constant, all other parameters are displayed in units of eV.

The modified Franck-Condon analysis of the samples with different concentrations is shown for each concentration in Figure S1. The complete set of parameters acquired is shown in TableS1. The *effective* non-aggregate spectra are acquired by subtracting the simulated aggregate spectra from the measured one. It is worth mentioning that the absorption spectra of push-pull polymers have a camel-back feature, where the absorbance between the high and low energy band is not completely zero due to the strong FE/CT mixing.[1] The oscillator strength ratios of the non-aggregate and aggregates absorption spectra are plotted in Figure S1(f).

The PL spectra are fitted with a single Gaussian distribution as shown in Figure S2. By subtracting the measurements with the Gaussian fit, a high-energy peak is observed when the samples are prepared from concentrations surpassing the critical chain overlap point.

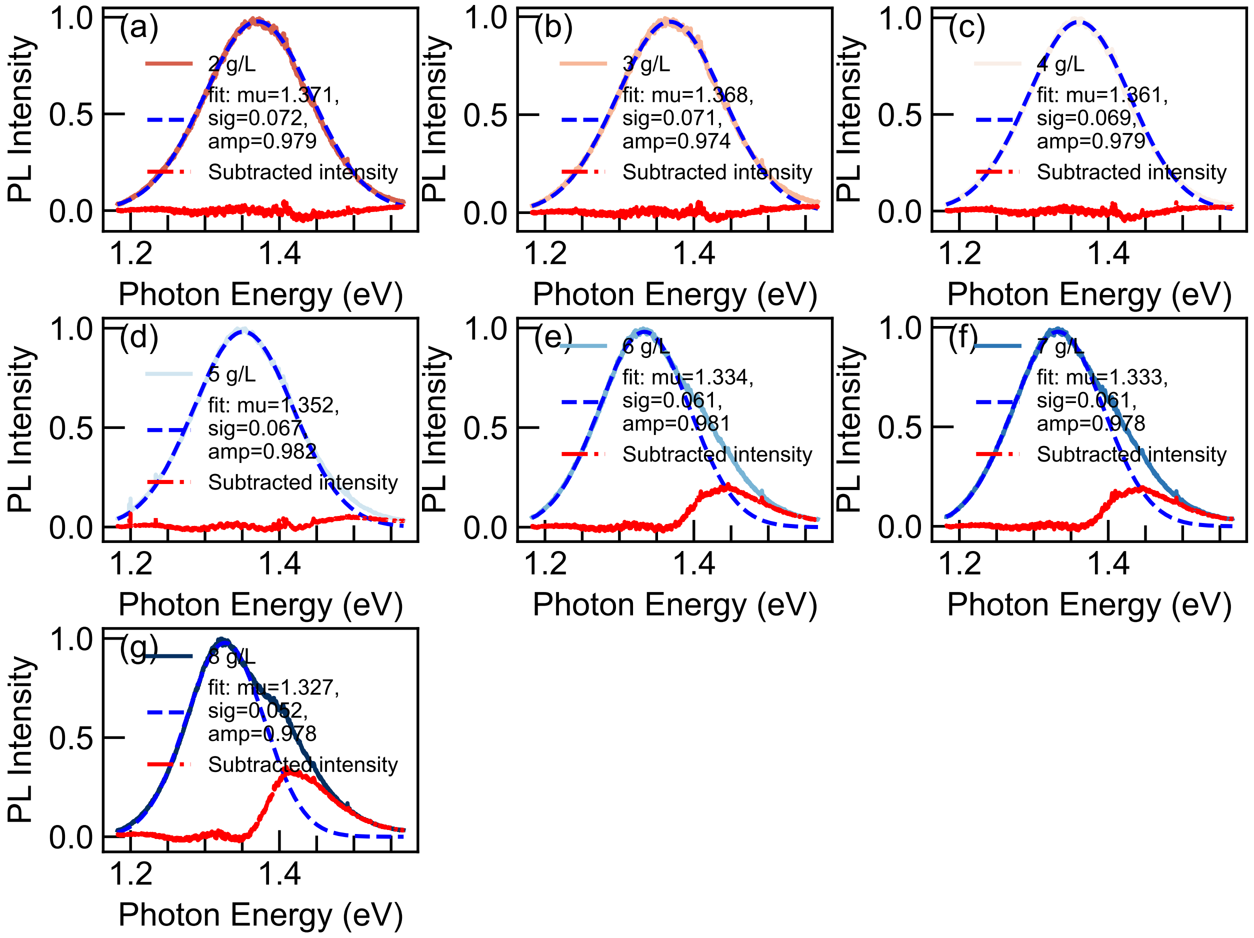

Figure S2: The Gaussian fit (blue dashed line) for PL spectra of thin film samples prepared from each concentration. The high energy shoulder is indicated by the red line.

Table S1: Parameters Acquired from Modified FC Fit

Conc. (g/L)	σ (meV)	E_0 (meV)	W (meV)	E_p (meV)	Cons.
2	71.9 ± 0.3	1495 ± 0.3	17.2 ± 0.6	167.0 ± 0.6	2.531 ± 0.006
3	75.7 ± 0.2	1492 ± 0.1	21.2 ± 0.3	171.0 ± 0.5	2.437 ± 0.002
4	75.9 ± 0.2	1495 ± 0.1	23.3 ± 0.3	170.1 ± 0.4	2.405 ± 0.002
5	78.5 ± 0.1	1500 ± 0.1	40.0 ± 0.2	173.9 ± 0.3	2.306 ± 0.001
6	73.9 ± 0.2	1508 ± 0.2	47.0 ± 0.3	164.7 ± 0.4	2.279 ± 0.002
7	69.4 ± 0.2	1512 ± 0.2	49.4 ± 0.3	156.5 ± 0.4	2.290 ± 0.003
8	70.7 ± 0.2	1511 ± 0.2	53.9 ± 0.4	159.4 ± 0.5	2.272 ± 0.003

From left to right are the widths of Gaussian inhomogeneous distribution used to describe the energetic disorder, σ; the energy of the 0-0 vibronic transition, E_0; exciton bandwidth, W; the energy of the vibrational mode coupled to the electronic transition, E_p and the fit constant.

Differential Scanning Calorimetry

The differential scanning calorimetry is conducted with TA Instruments DSC250. The thermograph is shown in Figure S3. With a cooling and heating rate of 20 °C/min, no meaningful differences are observed with samples prepared from 2 to 8 g/L. All heating curves display melting peaks at 375±2 °C with endothermic enthalpy of 32±4 °C.

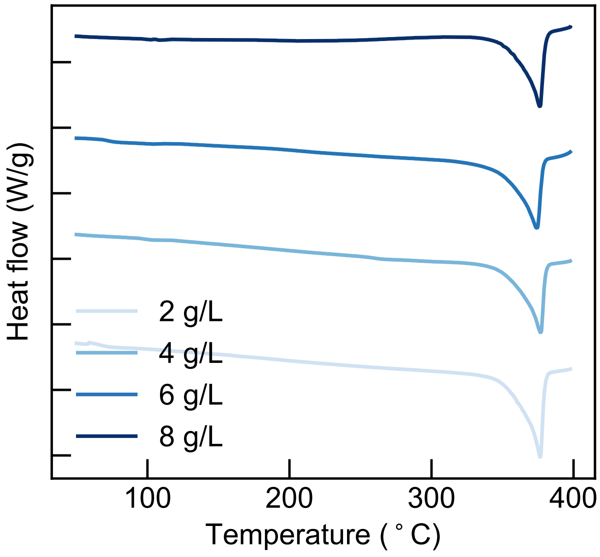

Figure S3: First heating curves of drop-cast films prepared from concentrations of 2, 4, 6 and 8 g/L with endo-down heating flow.

Table S2: DPP-DTT Thermal Properties

Conc. (g/L)	enthalpy (J/g)	onset temp. (°C)	peak temp. (°C)
2	32	364	377
4	29	362	377
6	36	356	374
8	29	362	376

The first heating DSC thermogram for DPP-DTT thin films precipitated from solution concentrations of 2, 4, 6 and 8 g/L. The heating and cooling rate is 20 °C/min.

Transient absorption

The transient absorption spectra measured under the lowest (Fig. S4) and highest pump fluences (Figure S6) that are feasible for clear signals, respectively. The fluences are displayed in each spectrum. We also took the temporal cuts at 1, 10, 100 and 800 ps time delays pumped at low fluences as shown in Figure S5. Due to the relative low fluences, signal-to-noise ratio at time delays beyond 100 ps are relatively low. It might be one of the reasons for the unusual feature in Figure 3 in the main article.

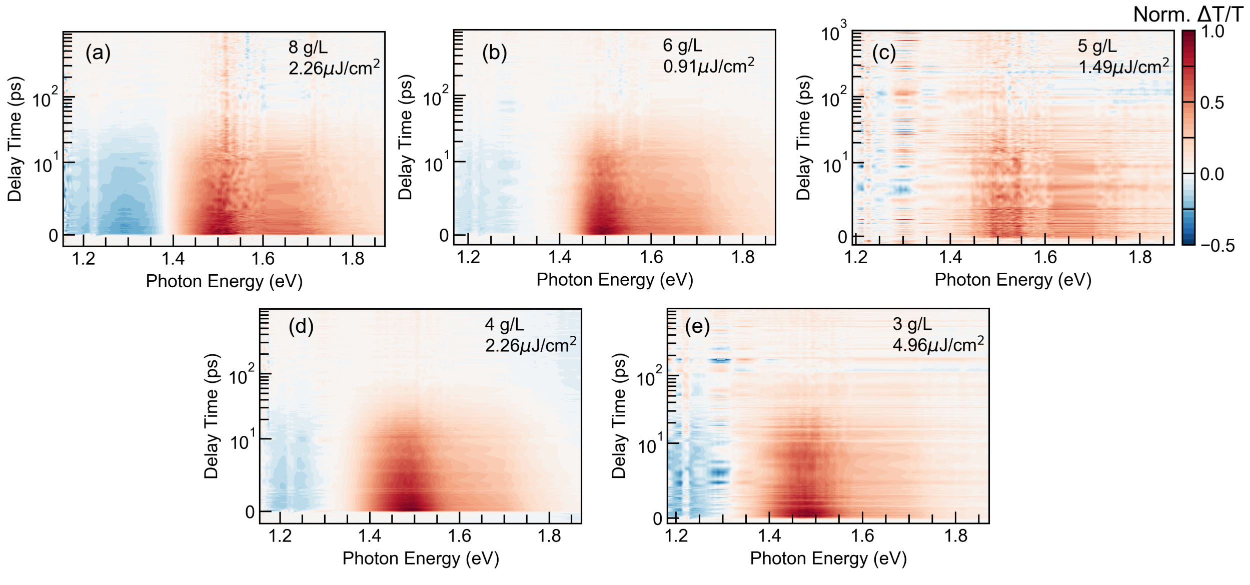

Figure S4: Normalized transient absorption time-energy map collected at the lowest pump fluences for 3, 4, 5, 6 and 8 g/L. The fluences are indicated in the annotation.

For the purpose of demonstration here, the transient absorption decays at 750 and 950

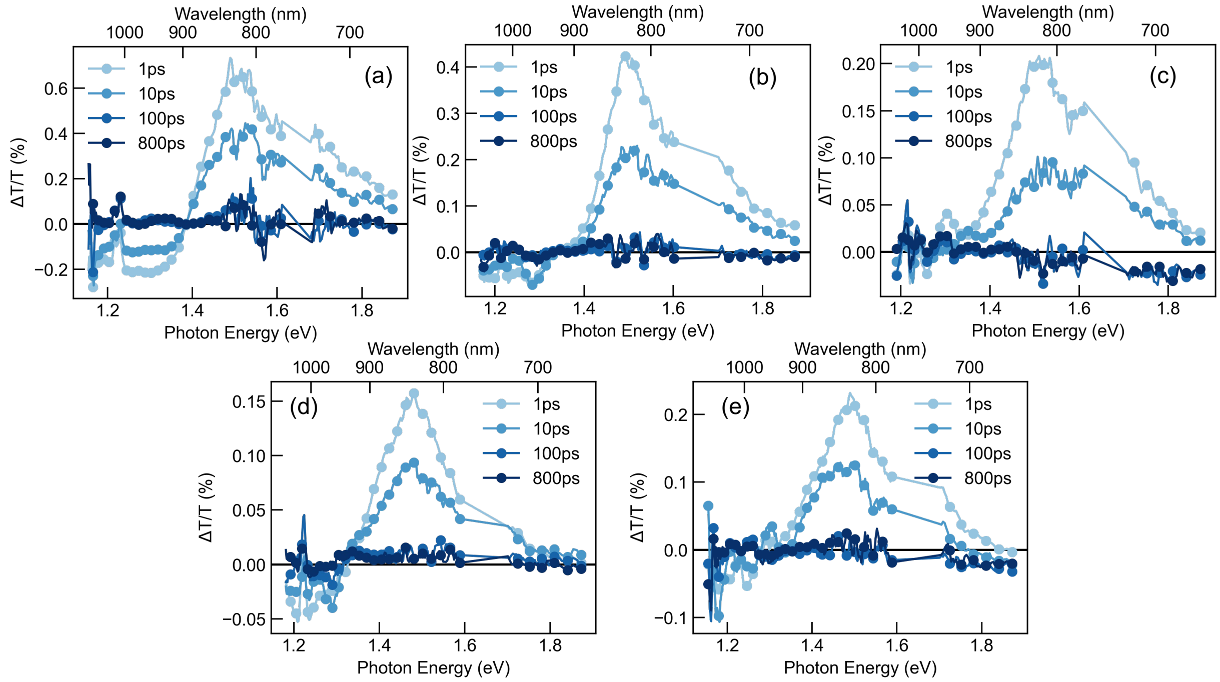

Figure S5: The spectra cuts taken at varying time delays. (a), (b), (c), (d), and (e) are the spectra of 8, 6, 5, 4, and 3 g/L, respectively. The corresponding fluences are shown in Figure S4

nm for 8 g/L are displayed in Figure S7. Although not directly shown here, the samples of other concentrations show similar dynamics. At the fluence of 2.2 μJ/cm^2, the apparent monoexponential lifetime is around 18 ps, where we assume the exciton-exciton annihilation is not significant at such low fluence.

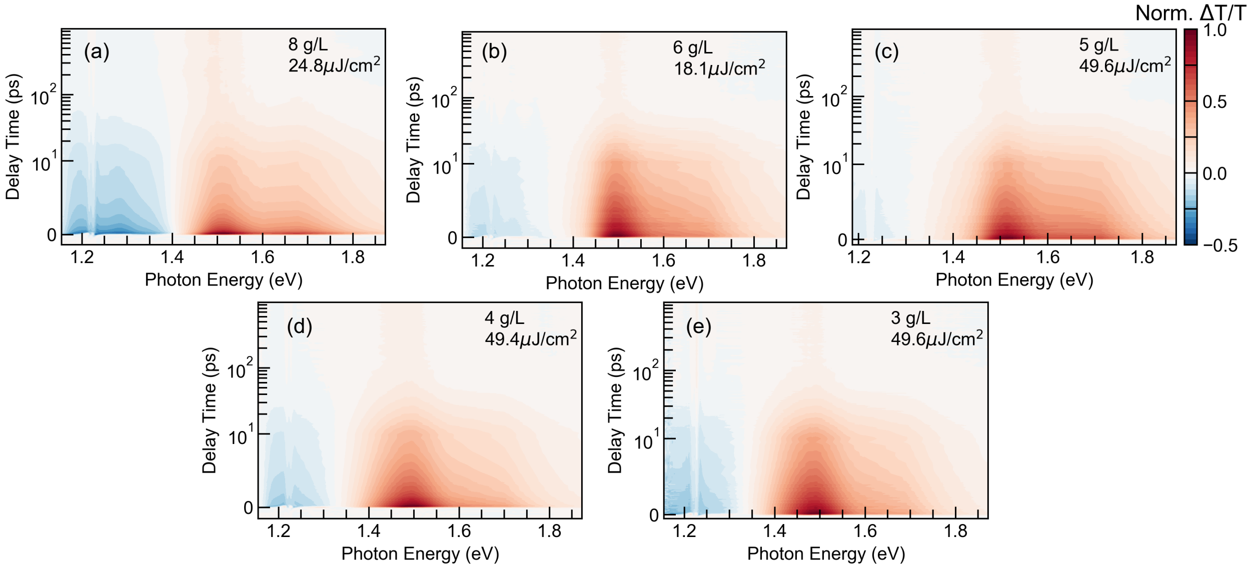

Figure S6: Normalized transient absorption time-energy map collected at the highest pump fluences for 3, 4, 5, 6 and 8 g/L. The fluences are indicated in the annotation.

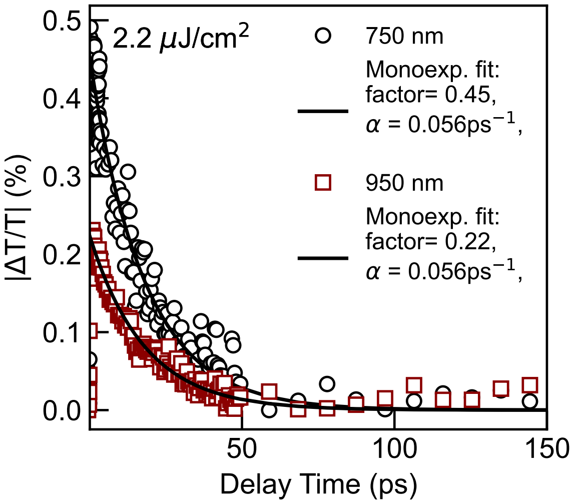

Figure S7: The TA decays at 750 nm (black circle) and 950 nm (brown square), respectively. in the 8g/L film. The solid lines display the monoexponential fit.

Quantum Chemistry Calculations

Density functional theory (DFT) and time-dependent DFT (TDDFT) calculations were performed using the Gaussian 16 Rev. A.03 software suite.[2] A DPP-DTT trimer, terminated at both ends with thiophene-flanked DPP units for symmetry, was optimized at the LC-ωHPBE/6-311G(d) level of theory. Empirical gap tuning was performed for the two oligomer geometries shown in Figure S8 following the method of Sun et al.,[3–7] obtaining converged range-separation parameters of $\omega_1 = 0.1295$ for geometry 1 (G1) and $\omega_2 = 0.1216$ for geometry 2 (G2). Alkyl side chains on DPP units were truncated to methyl units to reduce computational cost. Following optimization, vibrational frequency analysis was performed on the oligomer to verify that the obtained geometry was an energy minimum, as well as to obtain IR and Raman spectra and vibrational modes. Vibrational scaling factors of 0.995 (G1) and 0.968 (G2) were applied to the calculated Raman frequencies. The Raman activities were converted to intensities I_i using the scattering equation

$$I_i = \frac{(\nu_0 - \nu_i)^4 S_i}{\nu_i[1 - \exp(-\frac{hc\nu_i}{kT})]}$$

where ν_0 is the excitation wavelength; ν_i and S_i are the spatial frequency and Raman activity of vibrational mode i; and h, c, and k are Planck's constant, the speed of light, and Boltzmann's constant, respectively.[8,9] The temperature was taken to be 298.15 K. A TDDFT calculation was performed to obtain the excitation wavelength, 439 nm (22780 cm^{-1}), corresponding to the wavelength used in the associated experimental spectroscopy.

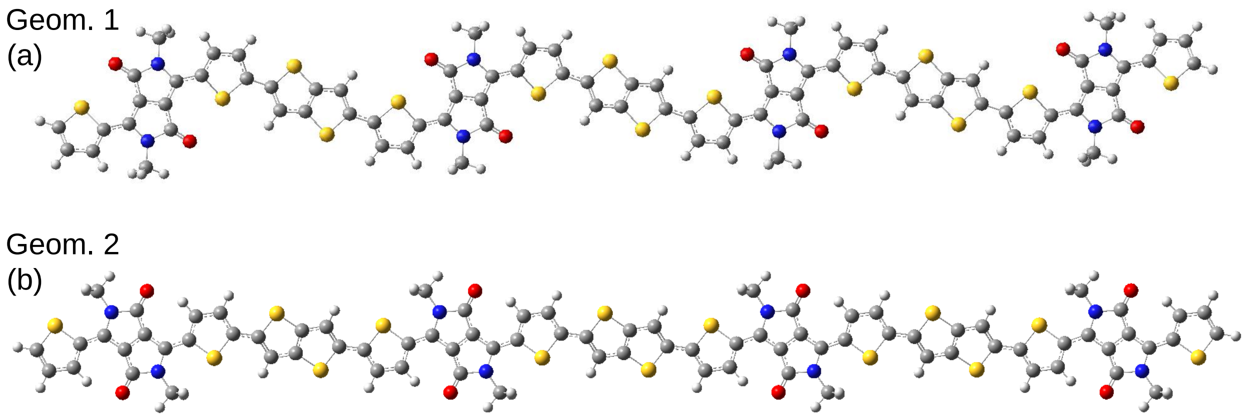

Figure S8: DFT-optimized molecular structures of (a) geometry 1 (G1) and (b) geometry 2 (G2). Notice that in G1, the oxygen atom in DPP unit is close to the sulfur atom in the neighboring thiophene unit, while in G2, the oxygen atom is in the vicinity of the hydrogen atom in the neighboring thiophene unit. Atomic color scheme: gray = carbon, white = hydrogen, yellow = sulfur, blue = nitrogen, red = oxygen.

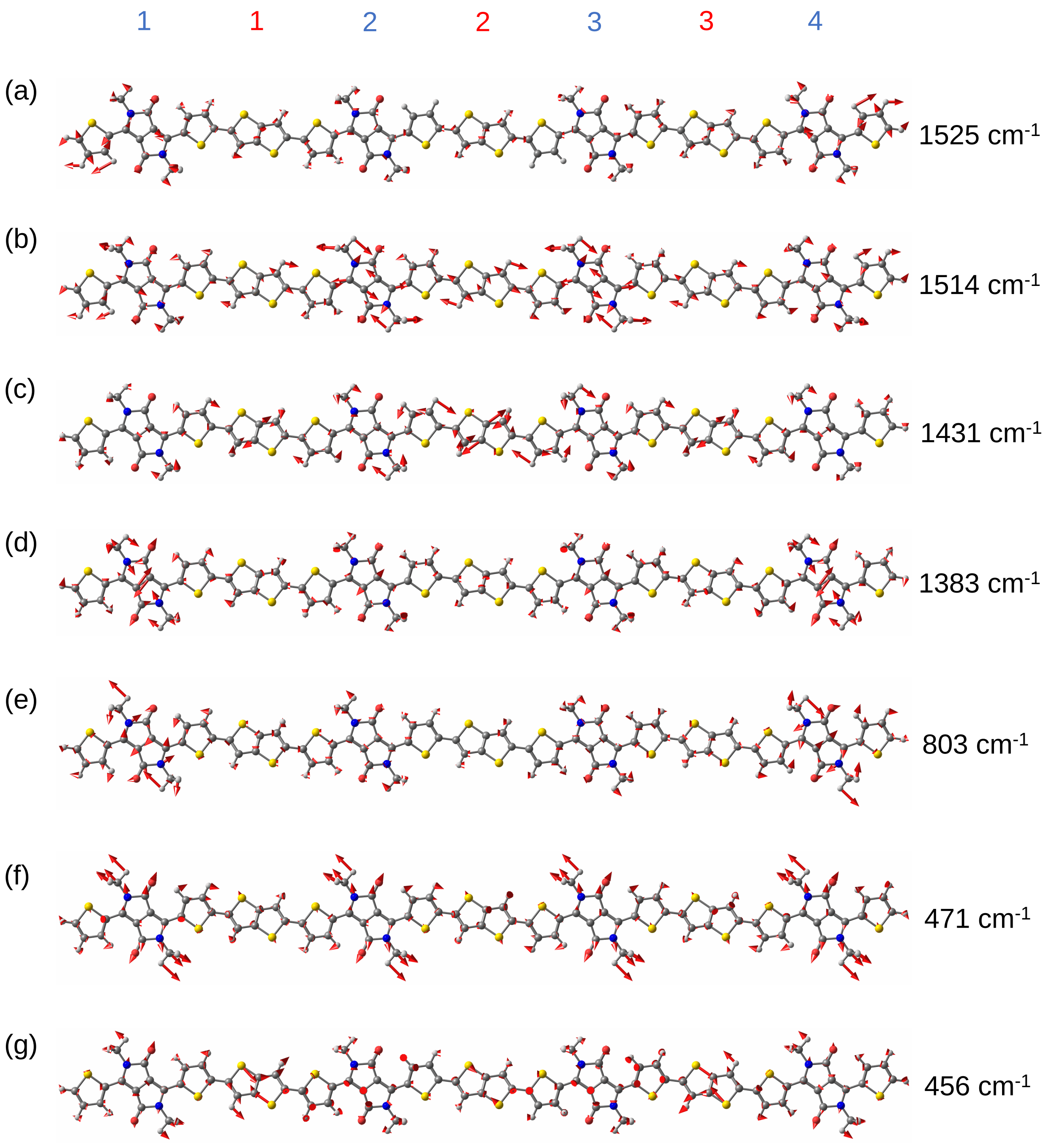

Figure S9: The vector diagrams for the modes of interest in geometry 2, corresponding to the geometry 1 modes shown in Figure 6 in the main article. The blue and red numbers at the top indicate the indices of the DPP and DTT units, respectively. Atomic displacements are indicated by the arrows.

References

(1) Banerji, N.; Gagnon, E.; Morgantini, P.-Y.; Valouch, S.; Mohebbi, A. R.; Seo, J.-H.; Leclerc, M.; Heeger, A. J. Breaking down the problem: optical transitions, electronic structure, and photoconductivity in conjugated polymer PCDTBT and in its separate building blocks. *The Journal of Physical Chemistry C* **2012**, *116*, 11456–11469.

(2) Frisch, M. J.; Trucks, G. W.; Schlegel, H. B.; Scuseria, G. E.; Robb, M. A.; Cheeseman, J. R.; Scalmani, G.; Barone, V.; Petersson, G. A.; Nakatsuji, H. et al. Gaussian˜16 Revision A.03. 2016; Gaussian Inc. Wallingford CT.

(3) Sun, H.; Ryno, S.; Zhong, C.; Ravva, M. K.; Sun, Z.; Körzdörfer, T.; Brédas, J.-L. Ionization Energies, Electron Affinities, and Polarization Energies of Organic Molecular Crystals: Quantitative Estimations from a Polarizable Continuum Model (PCM)-Tuned Range-Separated Density Functional Approach. *Journal of Chemical Theory and Computation* **2016**, *12*, 2906–2916.

(4) Henderson, T. M.; Izmaylov, A. F.; Scalmani, G.; Scuseria, G. E. Can short-range hybrids describe long-range-dependent properties? *The Journal of Chemical Physics* **2009**, *131*, 044108.

(5) Vreven, T.; Frisch, M. J.; Kudin, K. N.; Schlegel, H. B.; Morokuma, K. Geometry optimization with QM/MM methods II: Explicit quadratic coupling. *Molecular Physics* **2006**, *104*, 701–714.

(6) Vydrov, O. A.; Scuseria, G. E. Assessment of a long-range corrected hybrid functional. *The Journal of Chemical Physics* **2006**, *125*, 234109.

(7) Vydrov, O. A.; Scuseria, G. E.; Perdew, J. P. Tests of functionals for systems with fractional electron number. *The Journal of Chemical Physics* **2007**, *126*, 154109.

(8) Polavarapu, P. L. Ab initio vibrational Raman and Raman optical activity spectra. *The Journal of Physical Chemistry* **1990**, *94*, 8106–8112.

(9) Keresztury, G.; Holly, S.; Besenyei, G.; Varga, J.; Wang, A.; Durig, J. Vibrational spectra of monothiocarbamates-II. IR and Raman spectra, vibrational assignment, conformational analysis and ab initio calculations of S-methyl-N,N-dimethylthiocarbamate. *Spectrochimica Acta Part A: Molecular Spectroscopy* **1993**, *49*, 2007–2026.
